# A cross-sectional analysis of the relationship between tobacco and alcohol outlet density and neighbourhood deprivation

**DOI:** 10.1186/s12889-015-2321-1

**Published:** 2015-10-05

**Authors:** Niamh K Shortt, Catherine Tisch, Jamie Pearce, Richard Mitchell, Elizabeth A Richardson, Sarah Hill, Jeff Collin

**Affiliations:** Centre for Research on Environment, Society and Health, School of Geosciences, University of Edinburgh, Drummond St, Edinburgh, EH8 9XP Scotland, UK; Centre for Research on Environment, Society and Health, Institute for Health and Wellbeing, University of Glasgow, 1 Lilybank Gardens, Glasgow, G12 8RZ Scotland, UK; Social Policy, School of Social and Political Science, University of Edinburgh, 15a George Square, Edinburgh, EH8 9LD Scotland, UK

**Keywords:** Alcohol, Tobacco, Health inequalities, Retail environment, Density, Deprivation

## Abstract

**Background:**

There is a strong socio-economic gradient in both tobacco-and alcohol-related harm. One possible factor contributing to this social gradient may be greater availability of tobacco and alcohol in more socially-deprived areas. A higher density of tobacco and alcohol outlets is not only likely to increase supply but also to raise awareness of tobacco/alcohol brands, create a competitive local market that reduces product costs, and influence local social norms relating to tobacco and alcohol consumption. This paper examines the association between the density of alcohol and tobacco outlets and neighbourhood-level income deprivation.

**Methods:**

Using a national tobacco retailer register and alcohol licensing data this paper calculates the density of alcohol and tobacco retail outlets per 10,000 population for small neighbourhoods across the whole of Scotland. Average outlet density was calculated for neighbourhoods grouped by their level of income deprivation. Associations between outlet density and deprivation were analysed using one way analysis of variance.

**Results:**

There was a positive linear relationship between neighbourhood deprivation and outlets for both tobacco (*p* <0.001) and off-sales alcohol (*p* <0.001); the most deprived quintile of neighbourhoods had the highest densities of both. In contrast, the least deprived quintile had the lowest density of tobacco and both off-sales and on-sales alcohol outlets.

**Conclusions:**

The social gradient evident in alcohol and tobacco supply may be a contributing factor to the social gradient in alcohol- and tobacco-related disease. Policymakers should consider such gradients when creating tobacco and alcohol control policies. The potential contribution to public health, and health inequalities, of reducing the physical availability of both alcohol and tobacco products should be examined in developing broader supply-side interventions.

## Background

Tobacco and alcohol use continue to pose significant public health challenges and are leading causes of preventable morbidity and mortality worldwide [1, 2]. Combined tobacco-and alcohol-related illnesses are estimated to account for 12.5 % of all deaths globally [1]. Strong socio-economic gradients in consumption of, and harm from, both substances persist. Smoking and heavy alcohol consumption are inextricably linked to poverty and deprivation [3]. Research has shown that socially deprived populations are more likely to report heavier drinking [4], and to die from alcohol-related causes [5, 6]. Furthermore smoking rates have been declining at a faster pace amongst higher compared to lower socio-economic groups [7].

Alcohol-and tobacco-related behaviours are strongly influenced by a multitude of social, cultural and environmental factors [8]. One possible factor contributing to the social gradient in tobacco-and alcohol-related harm may be greater availability of tobacco and alcohol in more socially-deprived areas. A higher density of tobacco and alcohol outlets is not only likely to increase supply but also to raise awareness of tobacco/alcohol brands, create a competitive local market that reduces product costs, and influence local social norms relating to tobacco and alcohol consumption [9]. Research, largely from North America, has shown that tobacco and alcohol outlets are more prevalent in deprived areas and are independently associated with higher likelihood of smoking and drinking [10–16]. In response to such evidence the Institute of Medicine has called for a restriction on the number of tobacco outlets [17] while the World Health Organisation recommends national-level action plans that regulate the availability of alcohol [2]. Such supply side interventions may represent a new direction in both tobacco and alcohol control.

To date, research has tended to explore smoking and alcohol environments separately. This is problematic since alcohol and tobacco outlets often co-locate, and evidence suggests that related behaviours also co-occur [18]. This paper, focussing on neighbourhoods in Scotland, UK, is the first to investigate whether more socially-deprived areas tend also to have greater availability of both alcohol and tobacco outlets. Combined, smoking and alcohol intake are two of the most important preventable causes of ill-health and premature death in Scotland, where one in every five deaths is attributable to tobacco [19] and one in 20 attributable to alcohol [20]. Such deaths and ill-health have a marked social gradient. In Scotland those living in the most deprived neighbourhoods are 6 times more likely to die from an alcohol-related illness, and 7.5 times more likely to be hospitalised for an alcohol-related illness compared to those in the least deprived neighbourhoods [21]. Similarly 32 % of deaths in the most deprived areas of Scotland are attributable to smoking, compared with 15 % in more affluent areas [22], with smoking rates ranging from 40 % in the former to 10 % in the latter [23]. Such trends in alcohol and tobacco related health are not unique to Scotland and as such alcohol and tobacco consumption may be key factors in understanding the persistence of national level health inequalities over recent decades, with both factors on the pathway between social disadvantage and poor health [24].

## Methods

### Outlet data

The Tobacco and Primary Medical Services (Scotland) Act 2010 established a national register requiring all Scottish retailers selling tobacco products to be registered by 1 October 2011. We obtained the addresses and postcodes of all premises registered on the Scottish Tobacco Retailers Register as at 30 September 2012 (*n* = 11,449). After removing duplicates our final dataset contained 10,161 tobacco outlets.

All premises selling alcohol in Scotland must be licensed under the Licensing (Scotland) Act 2005. We obtained the addresses and postcodes of outlets licensed to sell alcohol on site, such as a restaurant or bar (‘on-sales’ *n* = 11,359) and for those licensed to sell alcohol for consumption off the premises (‘off-sales’ *n* = 4,800) in 2012 from individual local Liquor Licensing Boards (*n* = 36). The format of the data varied (some as lists in word documents, others as excel spread sheets) with the data collection stage taking 9 months. We checked the number of premises in our dataset against official published statistics. Our outlet dataset (collected in Autumn 2012) had 1.3 % fewer on-sales, 1.4 % fewer off-sales, and 1.4 % fewer outlets overall than reported by the Scottish Liquor Licensing Statistics 2011–12 (as of 31 March 2012) [25]. Part of the discrepancy could be due to our data collection later in 2012, and part due to our careful cleaning of the dataset to remove duplicate entries from the licensing board data we were provided with.

We created measures of outlet density for every data zone in Scotland with population (*n* = 6,502), using ESRI ArcMap 10.1 geographical information system (GIS) software. Data zones are the core small area units in Scotland for which statistics are made available (mean population 817 in 2012, source: Information Services Division). First, we mapped locations of all tobacco and alcohol outlets based on the coordinates of their postcodes (each postcode in the UK represent approximately address points). We then undertook a Kernel Density Estimation (KDE). This transforms the spatial pattern of outlet locations into a continuous ‘surface’ which represents the density of outlets and is not constrained by area-level boundaries. In brief, the KDE process divides Scotland into 100x100 m grid cells, and assesses the number and proximity of outlets within an 800 m radius for each cell. This radius was chosen as a plausible walking distance to get to an outlet. Outlets nearer the centre of the search window are given greater weight than those further away. As a result, rather than reporting the number of outlets for each data zone, the KDE value represents a proximity-weighted estimate of the density of each outlet type per km^2^. This method has advantages over other density measures as it considers density and proximity together [26]. We created KDE surfaces for all tobacco outlets, all alcohol outlets, alcohol off-sales outlets and alcohol on-sales outlets. We assigned each data zone the KDE values for the cell in which its population-weighted centroid was located, rather than the data zone mean, to better reflect the density of outlets where the majority of population reside.

### Neighbourhood deprivation

We then gathered an indicator of socio-economic deprivation for each data zone. The most appropriate indicator was a proxy for income deprivation sourced from the Scottish Government’s Scottish Index of Multiple Deprivation (SIMD) 2012. The SIMD is the Scottish Government’s tool for measuring deprivation at a local area level. As an area based measure the SIMD can tell us how different areas compare to one another. The SIMD consists of 7 domains ranging from education to crime. We chose not to use the overall measure of the SIMD (which includes all 7 domains) as the access domain included drive times to petrol stations which may sell tobacco and alcohol products). Following previous precedent, we selected the income deprivation domain as our measure of area level deprivation [24, 25]. This domain measures the proportion of the population in each area experiencing income deprivation as measured by receipt of means-tested benefits and support from the government (Table 1). Eligibility for means tested benefits is based on the amount of income and savings an individual has, as such benefits are meant to top-up income if it is below a certain level. A research ethics review was carried out and approved by the School of Geosciences, University of Edinburgh’s research ethics committee. The study analysed census data at an area unit level and as such written consent from individuals was not required.

**Table 1:** Benefits included in income deprivation domain of the Scottish index of multiple deprivation 2012

Households included in Income Deprivation Domain
Adults and Children in Income Support (IS) Households
Adults and Children in Job Seekers Allowance (JSA) households
Adults in Guarantee Pension Credit Households
Adults and Children in Tax Credit Households on low incomes

### Analysis

We used two complementary approaches to assess the relationship between outlet density and income deprivation, because each was better at highlighting particular features of these complex relationships.

First, the data zones were divided into quintiles of income deprivation (1 = least deprived, 5 = most deprived) based on the income deprivation rank, generated from the percentage of income deprived population in each data zone (Table 2). Such quintiles are used extensively in policy related research in the Scottish Government. Outlet density rates per 10,000 total population were then calculated for each income deprivation quintile, and compared using one-way analysis of variance (ANOVA). Analysis were performed in STATA /IC 12.0.

**Table 2:** Proportion of households receiving means-tested benefits in each data zone quintile of income deprivation

Income deprivation quintiles	Proportion of data zone population receiving means-tested benefits^a^	Income deprivation rank^b^	Total data zones in each quintile	Total households in each quintile
1 (least deprived)	0–5 %	5203–6502	1300	454916
2	5–9 %	3903–5202	1300	512592
3	9–14 %	2602–3902	1301	516418
4	14–22 %	1302–2601	1300	509263
5 (most deprived)	22–65 %	1–1301	1301	526884

^a^due to anonymity agreements, proportion of households receiving means-tested benefits is rounded to the nearest whole percentage for publication

^b^unrounded rates were used to calculate the income domain rank

Second, we treated both neighbourhood income deprivation (proportion of households receiving means-tested benefits) and the density values (outlets per km^2^) as continuous measures. This enabled us to examine variations in outlet density within neighbourhood income deprivation quintiles. We calculated population-weighted outlet density values for each income deprivation percentage point (i.e. additional percentage of households receiving means-tested benefits in a given area) using the following equation:

$$ KD{E}_i=\frac{{\displaystyle {\sum}_{j=1}^n}\left(KD{E}_j\times po{p}_j\right)}{{\displaystyle {\sum}_{j=1}^n}\left(po{p}_j\right)} $$

where for each income deprivation percentage point *i*, *KDE*_*i*_ is the population-weighted KDE value, *n* is the total number of data zones within i, and KDE_j_ and pop_j_ are the density value and population, respectively, for the jth data zone within i. These values were then plotted on a bubble graph, with symbol size proportional to the number of data zones at each percentage point. We used this approach to explore the location of alcohol and tobacco outlets by data zone income deprivation. Finally, we also used this method to examine the co-location of *both* alcohol and tobacco outlets by neighbourhood income deprivation.

## Results

**Fig. 1:**
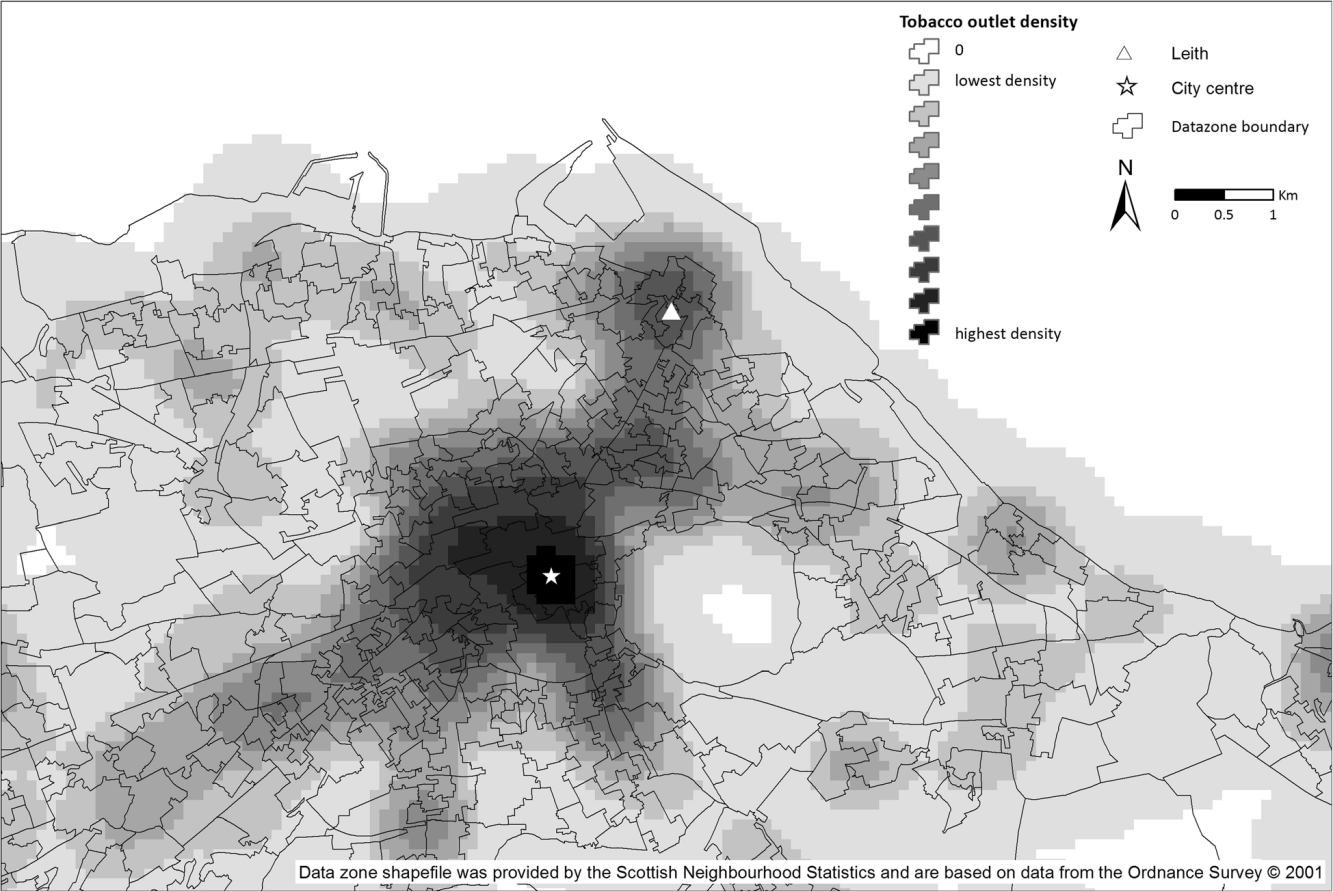
Tobacco outlet density across Edinburgh. Copyright of underlying shapefiles held by Ordnance Survey. Permission to use and publish these files given under Digimap End Users Licencing Agreement Clause 3.3.8

Figure 1 shows an example tobacco outlet density surface, for Edinburgh. Unsurprisingly density was highest in the city centre but there are also pockets of high density in other parts of the city including to the north (Leith).

For all types of outlet there was a statistically significant difference (*p* = <0.001) in outlet density between income deprivation quintile groups (quintile 1 = least deprived, quintile 5 = most deprived) (Table 3). The average density of tobacco outlets increased from 49.6 per 10,000 population in the least income deprived areas to 99.9 per 10,000 in the most deprived areas, with a linear increase across the quintiles (*p* <0.001). The density of outlets in the most deprived areas (quintile 4 and quintile 5) was significantly higher than in the less deprived areas (quintile 1 and quintile 2). For total alcohol outlets the least deprived areas (quintile 1 and quintile 2) again had significantly lower outlet densities compared to the most deprived areas (quintile 5), but the highest density was found for data zones at a medium level of deprivation (quintile 3).

**Table 3:** Mean tobacco and alcohol outlet densities (proximity-weighted) per 10,000 population in each income deprivation quintile

Income deprivation quintiles	Tobacco outlets per 10,000 population (95 % CIs)	Total Alcohol outlets per 10,000 population (95 % CIs)	Off-sales alcohol outlets per 10,000 population (95 % CIs)	On-sales alcohol outlets per 10,000 population (95 % CIs)
1 (least deprived)	49.6 (44.2–54.9)	84.7 (73.1–96.3)	25.0 (22.7–27.3)	59.7 (50.0–69.3)
2	64.3 (56.1–72.5)	106.8 (92.3–121.3)	30.4 (26.3–34.5)	76.4 (65.2–87.6)
3	86.1 (79.7–92.6)	129.8 (117.9–141.7)	40.2 (37.4–43.0)	89.6 (79.7–99.4)
4	94.6 (89.7–99.5)	128.5 (119.7–137.3)	46.6 (44.4–48.7)	82.0 (74.8–89.1)
5 (most deprived)	99.9 (95.1–104.7)	122.4 (114.9–129.9)	52.9 (50.8–54.9)	69.6 (63.6–75.5)
*P* value (ANOVA)	<0.001	<0.001	<0.001	<0.001

Exploring alcohol outlet density by type (off-sales and on-sales) revealed different patterns. The density of off-sales outlets was significantly higher in the most deprived areas (52.9 95 % CI 50.8–54.9) compared to all other quintiles, and off-sales outlet density increased linearly with deprivation. However, the highest density of on-sales outlets was found for data zones at a medium level of deprivation (quintile 3), similar to the total alcohol outlets distribution. The least deprived areas (quintile 1) consistently had the lowest density of alcohol and tobacco outlets.

**Fig. 2:**
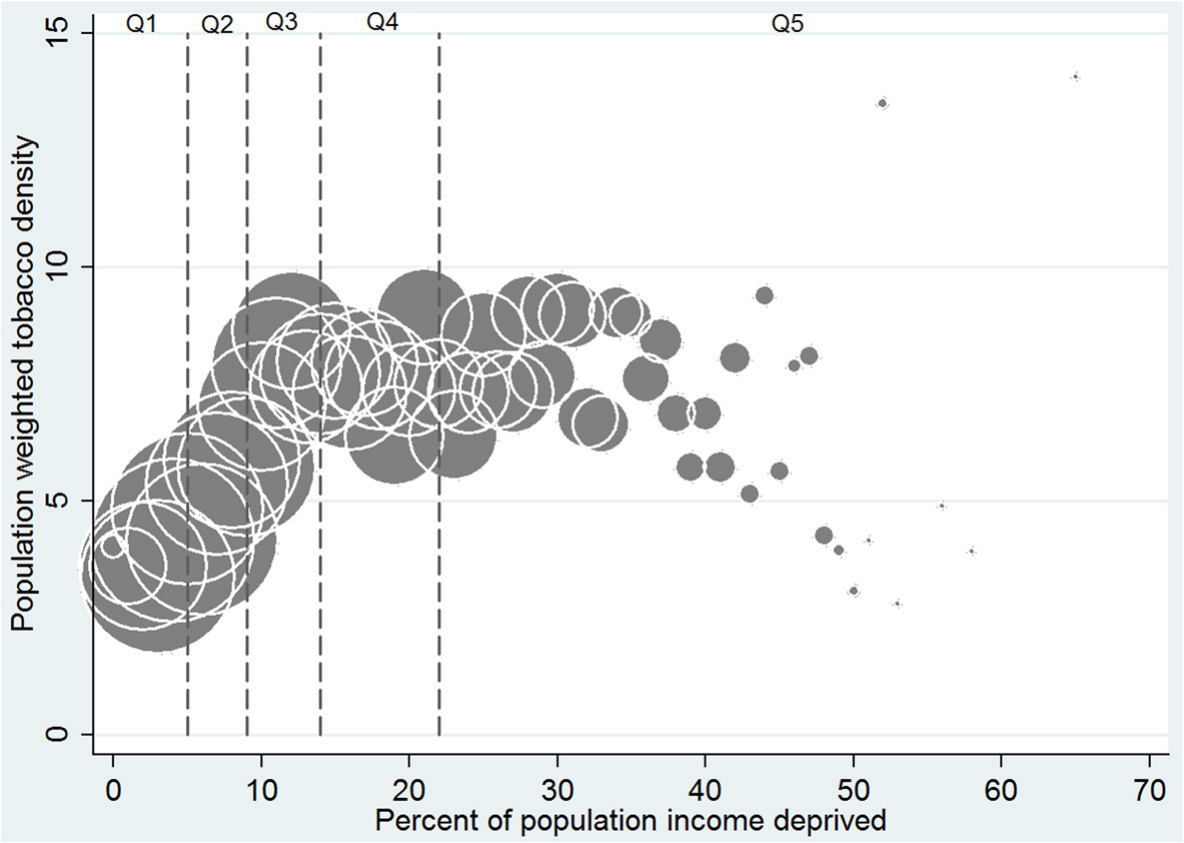
Tobacco outlet density by proportion of data zone population receiving means-tested benefits. Population weighted Kernel Density Estimates with bubble size proportional to the number of data zones (total number = 6502) represented by each point

**Fig. 3:**
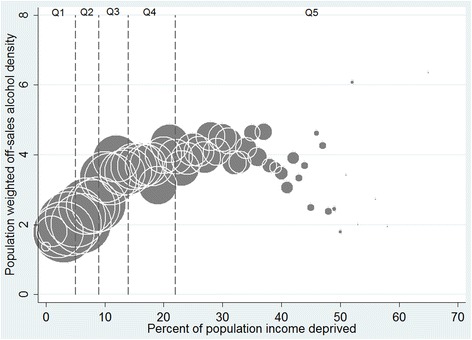
Off-sales alcohol outlet density by proportion of data zone population receiving means-tested benefits. Population weighted Kernel Density Estimates with bubble size proportional to the number of data zones (total number = 6502) represented by each point

These results mask the outlet density variation *within* each income deprivation quintile. We therefore explored this using income deprivation data on a continuous scale. As examples, Figs. 2 and 3 graph the density of tobacco and off-sales alcohol outlets for each additional percentage of households receiving means-tested benefits in each data zone, with the vertical lines on the graph representing quintiles of income deprivation. The density of tobacco and off-sales alcohol outlets increases as area-level income deprivation increases, but declines in areas of extremely high income deprivation. It should be noted that there are very few neighbourhoods at this end of the income deprivation scale: 105 data zones, or 1.6 % of Scotland’s 6505 data zones, have income deprivation rates of 40 % or above. The majority of data zones in the most income deprived quintile are in areas of highest outlet density (clustered between 23 % and 39 % income deprived).

**Fig. 4:**
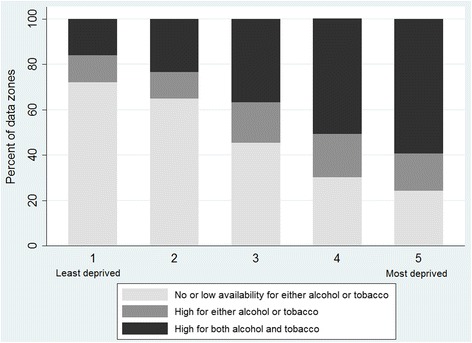
Availability of alcohol and tobacco outlets by neighbourhood income deprivation

Considering the co-location of alcohol and tobacco, a clear social gradient is evident in the distribution of high density areas by neighbourhood income deprivation (Fig. 4). Dividing the data into three groups of outlet availability allows us to see the spread of both alcohol and tobacco outlets across income deprivation quintiles. Those data zones with zero outlet density comprise one group (tobacco 541 datazones, total alcohol 460 datazones). The remaining two groups were equally divided based on their KDE scores (low availability ranged from 0.0001 to 4.20 for tobacco (*n* = 2982) and 0.0001 to 4.87 for alcohol (*n* = 3021), high availability from 4.21 to 122.15 for tobacco (*n* = 2979) and 4.88–255.50 for total alcohol (*n* = 3021). Across the whole of Scotland 37 % of neighbourhoods were in the highest group of availability in both tobacco and alcohol retailing combined, and an additional 15 % in the highest group of one or the other. There was evidence of strong social inequalities, with 59 % of the most income-deprived neighbourhoods in the highest group of both, compared with just 16 % of the least deprived neighbourhoods (Fig. 4).

## Discussion

In this paper we report a marked social gradient in both tobacco and alcohol off-sales retail outlets as well as in the *co-location* of these outlets, with higher densities of such retail outlets in more deprived areas and the lowest densities in the most affluent neighbourhoods. This is the first national level study that we are aware of that has simultaneously examined the relationship between neighbourhood deprivation and both alcohol and tobacco outlets, though patterns observed are similar to those reported separately for alcohol and tobacco in the USA [27, 28], New Zealand [29, 30] and Australia [31, 32]. Whilst the same broad pattern was *not* found for alcohol on-sales, in either this study or elsewhere [30, 32], it is important to note that recent research suggests that off-sales alcohol outlets have the greatest potential for alcohol related harm, in part due to cheaper products and competitive local markets [33]. Furthermore a rapidly growing off-sales trade, and the shifting spaces of alcohol consumption towards the home, suggest a need to consider the off sales trade in more detail.

We suggest that this gradient in alcohol and tobacco supply may be an important contributing factor in Scotland’s strong social gradient in alcohol-and tobacco-related disease. It seems likely that it will also contribute to such social gradients elsewhere in the economically developed world. This uneven distribution of negative retail and economic environments place the most disadvantaged populations in the most harm. Within this broad pattern, exploring the relationship between deprivation and outlet density on a continuous scale suggests a more complicated picture. Whilst the majority of those living in the most deprived data zones experience the highest retail densities, areas of extremely high-income deprivation have slightly lower outlet density. Neighbourhoods with extreme income deprivation (over 40 % of households receiving means-tested benefits) are likely to be multiply deprived, including a lack of access to basic resources such as retail stores, these neighbourhoods could be classed as ‘retail deserts’. Such a lack of general provision itself has important implications for well-being. Lower outlet densities in these areas may be linked to a lack of purchasing power in these communities. For both alcohol off-sales and tobacco, the norm is for such products to be sold alongside groceries and other household products in small stores or supermarkets. Such stores may simply not be viable in areas of extreme poverty. Nonetheless, evidence suggests that alcohol and tobacco related harm is highest in such areas suggesting that these populations may be more dependent upon contraband cigarettes [34, 35] and alcohol.

Smoking and alcohol intake are two of the main targets within global public health, but public and industry related rhetoric in these areas often frames them within libertarian arguments, with little or no recognition of the broader determinants of these ‘bad behaviours’ [36, 37]. Furthermore, the tobacco industry itself has appropriated and misrepresented the language of the social determinants model [38]. The social patterning of health behaviours reflects the socio-spatial patterning of these determinants [3, 4, 6, 8]. For tobacco and alcohol consumption, this includes the distribution of alcohol and tobacco retail outlets and the commercial drivers behind this distribution [39–41]. An improved understanding of such retail patterning may enhance our knowledge of the relationship between place and risky health behaviours, leading to a better understanding of what creates inequalities in such behaviours. If public health is serious about tackling tobacco and alcohol consumption as drivers of health inequalities then we need to consider the broader determinants of such behaviours, including the retail environment.

Our research has certain limitations. Whilst the tobacco outlet data was retrieved from the Scottish Tobacco Retailers Register, there is no central repository for comparable alcohol outlet data. As such we had to request the data from individual Scottish Licensing Boards. Whilst there may be an element of error we compared our data to aggregated national level official statistics which showed just 1.4 % fewer overall alcohol outlets in our dataset. This may be because we did not capture 100 % of outlets, but it may also be due to our careful cleaning of the data. This process was lengthy and to assist future research we would urge that the Government consider a central alcohol retailers register with further detail on shop floor and size of premise. In addition our dataset did not permit us to include opening hours in this analysis; such aspects of the environment may have particular relevance to city centre districts. Detail on premise size and hours would aid future, more detailed work in this area. A further limitation is the cross sectional nature of this analysis. We see this as the baseline for this data and in the future we plan to expand this work using longitudinal analysis.

Our findings suggest that the relationship between outlet density and prevalence of both tobacco and alcohol use merit more detailed exploration, and that such research has the potential to valuably inform policy development. Within tobacco control there has been increased recognition of the comparative neglect of the potential of supply side measures [42], and interventions to reduce the number of retail outlets have been identified as offering the prospect of a new frontier for policy innovation [43]. The potential contribution of restricting physical availability to reducing harm is perhaps more clearly recognised within alcohol policy [44], where “regulating the density of alcohol outlets and controlling the sales hours” are identified as policy options within WHO Europe’s action plan [2]. While our findings highlight that alcohol and tobacco outlets are most concentrated in more deprived neighbourhoods, it is worth noting that outlet densities are comparatively high in all areas. Even the most affluent neighbourhoods have high numbers of outlets, it is just that there are more in the most deprived neighbourhoods, but overall this over-abundance of supply exemplifys Cohen and Anglin’s reference to “a retail environment that practically spews cigarettes out of every crevice [43].”

Recent research has demonstrated associations between tobacco retail density and increased smoking in adolescence and adulthood, and between alcohol retail density and alcohol related mortality in the UK and beyond [26, 33, 45]. The possibilities created by the recently introduced Scottish Tobacco Retail Register (Scottish Parliament 2010) have been understood primarily with reference to enforcement of underage sales and display bans. However, a national register such as this offers enormous research potential to track change in the environment “with a view to considering further steps to regulate the supply of cigarettes” [46]. In contrast the difficulties we encountered gathering the alcohol data lead us to call for such national registers to become commonplace, for both alcohol and tobacco.

## Conclusions

Without a clear understanding of the social geography of availability and access, some supply-side interventions in tobacco and alcohol policy may inadvertently exacerbate inequities. Most recently the 2010 Scottish Tobacco Act’s point of sales display ban came into force with a staggered implementation beginning in larger retailers in 2013 and smaller shops in 2015. If comparatively fewer larger stores are located in more income deprived areas then early impacts of the ban could exacerbate inequalities in tobacco use, given the delay in implementation for small shops. Furthermore, liquor licensing boards in Scotland, who are charged with granting or refusing applications for licences to sell alcohol, are required to assess overprovision within a board’s areas. A distinct lack of data makes assessing such overprovision a near impossible task. Tobacco and alcohol control policies in Scotland since devolution in 1998 have been characterised by impressive levels of innovation, from the introduction of smoke-free public places, through the commitment to minimum unit pricing for alcohol, to the ambition to reduce adult smoking prevalence to below 5 % by 2034 [46]. An improvement to a nation’s health of this magnitude requires policy-makers to make a renewed commitment to addressing health inequalities–a task requiring political will, innovative policy and good data. An improved awareness of the relationships between retail availability and consumption of alcohol and tobacco could make an invaluable contribution in developing effective supply-side interventions. An economic system that places business before public health will not only damage health, but may also exacerbate health inequalities.

## Abbreviations

ANOVA: Analysis of variance
KDE: Kernel density estimation
GIS: Geographical information systems
SIMD: Scottish index of multiple deprivation
